# A Biological Solution to a Bony Problem: Interposition Arthroplasty for Post-traumatic Patellofemoral Arthritis

**DOI:** 10.7759/cureus.91409

**Published:** 2025-09-01

**Authors:** Wan Lye Cheong, Khairil Anwar Ahmad Hanif, Siti Munira Seri Masran, Mohd Afiq Muhamed Fuad, Syahril Izwan Alias, Fahrudin Che-Hamzah

**Affiliations:** 1 Department of Orthopaedics, Faculty of Medicine and Health Sciences, Universiti Putra Malaysia, Serdang, MYS; 2 Department of Orthopaedics, Hospital Sultan Abdul Aziz Shah, Serdang, MYS

**Keywords:** interposition arthroplasty, joint-preserving surgery, patellofemoral arthritis, post-traumatic arthritis, synovium

## Abstract

Interposition arthroplasty is a joint-preserving surgical technique that involves the placement of biological or synthetic tissue between articulating surfaces to restore function and alleviate pain. Although rarely used in modern practice, it remains a valuable option in selected cases, particularly in young, active patients with complex post-traumatic joint pathology. We report the case of a man in his 40s who presented with persistent anterior knee pain and functional impairment due to isolated post-traumatic patellofemoral arthritis and a significant trochlear bone defect following distal femur fracture fixation. Imaging revealed extensive trochlear cartilage loss, bony irregularities, and a contained central defect. We performed an interposition arthroplasty utilising an ipsilateral suprapatellar synovial flap, in conjunction with bone grafting of the osseous defect, trochleaplasty, patelloplasty, and lateral patellar facetectomy. Postoperatively, the patient experienced substantial pain relief and functional recovery, maintained at two-year follow-up. This case demonstrates that interposition arthroplasty, though seldom employed today, can offer excellent outcomes in carefully selected patients with isolated patellofemoral arthritis.

## Introduction

Isolated patellofemoral joint osteoarthritis (PFJ OA) is a potentially debilitating condition characterised by anterior knee pain, difficulty with stair climbing, and reduced functional capacity. It affects up to 25% of individuals aged 20 years and older, either in isolation or alongside tibiofemoral involvement [1]. Isolated PFJ OA involves cartilage loss affecting the patella, the trochlea, or both, in the absence of tibiofemoral degeneration [2]. The patellofemoral joint endures substantial biomechanical stress, bearing forces up to 3.3 times body weight during stair climbing and as much as 7.8 times during deep knee flexion [3]. These high joint loads, compounded by malalignment or instability, can accelerate articular degeneration.

Conservative measures such as physiotherapy, activity modification, and weight reduction are typically first-line treatments, particularly for mild to moderate disease. In advanced cases unresponsive to nonoperative management, surgical options range from soft tissue realignment and cartilage restoration to partial or total knee arthroplasty (TKA). While TKA provides reliable outcomes, it is often excessive for younger, active individuals. Patellofemoral arthroplasty (PFA) offers a joint-sparing alternative but has historically yielded variable results, particularly in the context of complex post-traumatic deformities. Earlier implant design saw a low satisfactory rate of 45% and a survival rate of 48% at six years [4]. Newer generation implants showed improvement in survival rates, but still fall short of TKAs, with PFA revision rates of 18.1-35% [5].

Interposition arthroplasty is a seldom-utilised biological technique that involves placing a soft tissue graft between joint surfaces to form a functional pseudarthrosis. Although more commonly described in the elbow, shoulder, and carpometacarpal joints, its use in the patellofemoral joint is rare [6,7]. However, this technique remains valuable for select cases where conventional prosthetic options are limited, particularly in young patients with isolated joint degeneration and challenging bone morphology. This case report highlights a successful application of interposition arthroplasty using a suprapatellar synovial flap to biologically resurface a large trochlear defect in a patient with post-traumatic patellofemoral arthritis.

## Case presentation

A 42-year-old man sustained a closed fracture of the right distal femur following a motor vehicle accident two years prior. He underwent open reduction and internal fixation with a locking plate. The fracture subsequently united, and he was allowed full weight-bearing. However, his knee range of motion (ROM) remained limited to 5-30 degrees. One year postoperatively, he underwent a second procedure involving implant removal and arthroscopic adhesiolysis, which improved his ROM to 0-90 degrees and enabled independent ambulation. Despite the improved mobility, he continued to experience persistent anterior knee pain, particularly during stair ascent and descent, which significantly interfered with his ability to return to work.

On examination, two well-healed surgical scars were noted: one lateral scar over the distal thigh and another anterior scar extending toward the anterolateral aspect of the knee. Knee ROM was 0-90 degrees, with palpable crepitus throughout the arc of motion and a positive patellar grind test. Ligamentous stability was intact.

**Figure 1:**
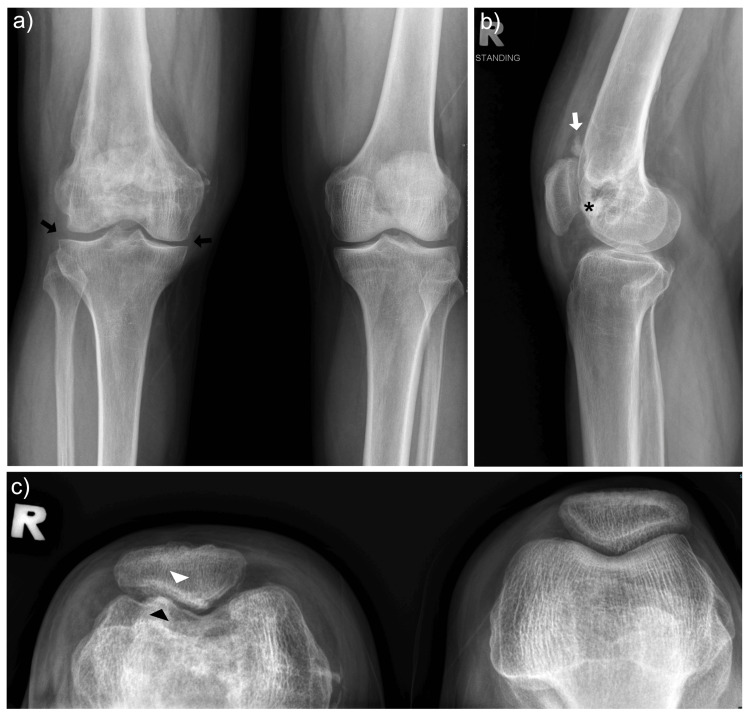
Pre-operative radiographs of the right knee (a) Standing anteroposterior (AP) view showing preserved medial and lateral tibiofemoral joint spaces (black arrow) (b) Lateral view demonstrating irregularity at the patellofemoral articulation (asterisk) and bone fragment (white arrow) within the joint space (c) Merchant view revealing patellofemoral arthritis characterised by subchondral sclerosis (white arrowhead) and surface irregularity (black arrowhead) over both the patella and trochlea

Plain radiographs revealed joint space narrowing over the lateral patellar facet, subchondral sclerosis, and a suspected bony defect in the trochlear region (Figure 1).

**Figure 2:**
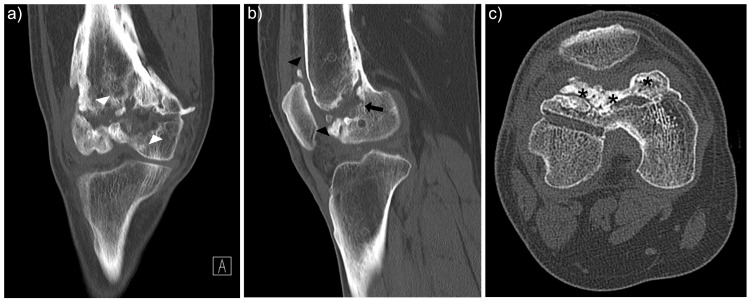
Pre-operative computed tomography (CT) images of the right knee (a) Coronal view demonstrating irregularity and bone loss (white arrowhead) over the anterior distal femur (b) Sagittal view showing significant trochlear surface irregularity, anterior distal femur bone loss (black arrow) and multiple intra-articular bone fragments (black arrowhead) within the patellofemoral compartment (c) Axial view illustrating patellofemoral arthritis with subchondral sclerosis and irregular articular surface (asterisk) over the trochlear groove

Computed tomography (CT) confirmed a contained trochlear defect measuring 5.1 cm × 1.2 cm × 2.6 cm with irregular lateral trochlear morphology (Figure 2).

He was planned for an interposition arthroplasty and bone grafting, which involved a medial parapatellar approach through the previous anterior incision. Intra-operatively, the suprapatellar synovium was markedly thickened. The trochlear surface was irregular with widespread cartilage loss, and a central bony defect filled with fibrous tissue was identified. This tissue was excised, and the defect was debrided to healthy bleeding bone before being filled with demineralised bone matrix. Trochleoplasty was performed to smooth the articular surface. The patella exhibited osteophytes and patchy chondral damage, which were addressed with patelloplasty and lateral patellar facetectomy.

**Figure 3:**
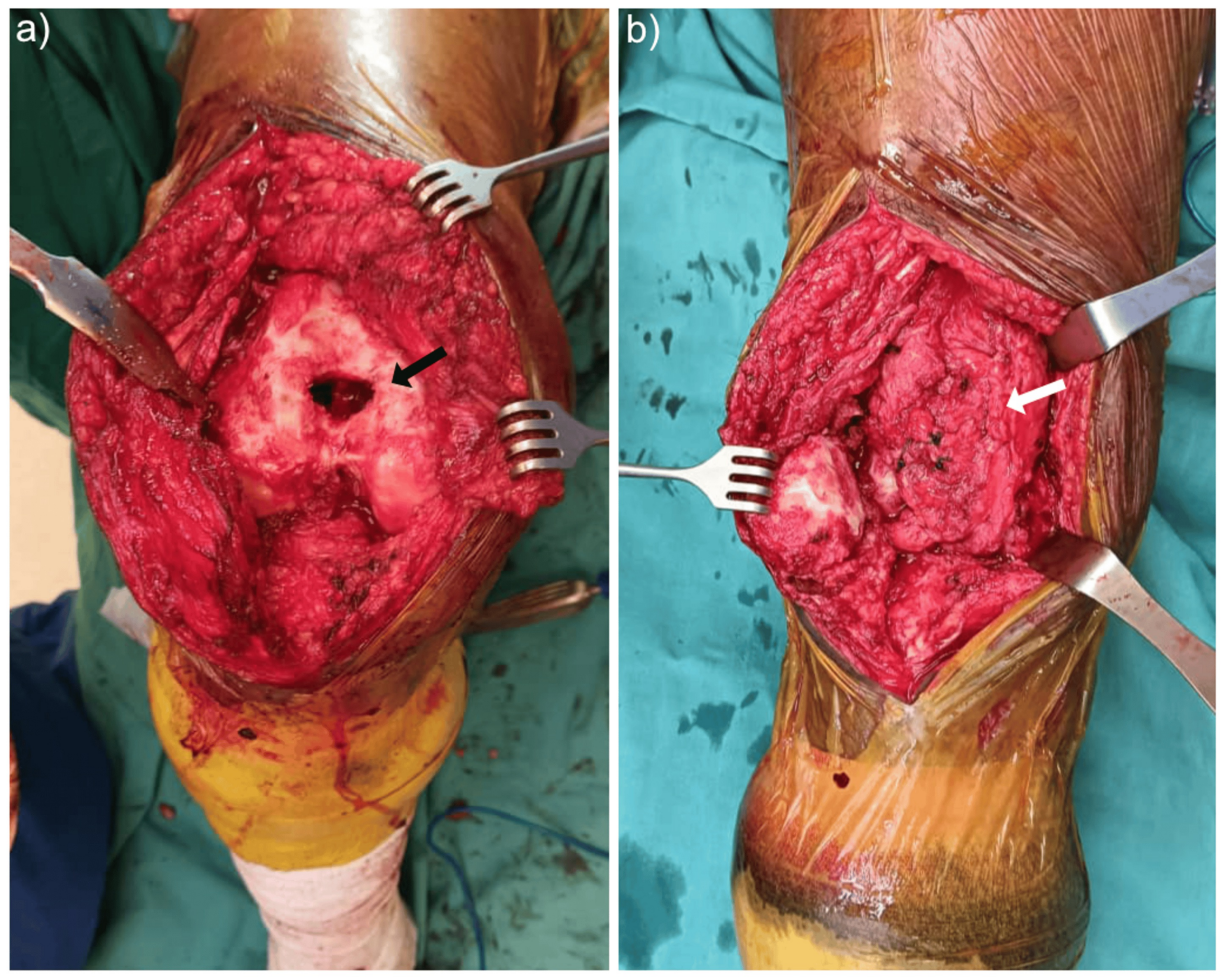
Intraoperative photos of the right knee (a) Exposed anterior surface of the distal femur after trochleaplasty with a bone defect (black arrow) seen (b) Suprapatellar synovium flap (white arrow) layered onto the trochlea

A proximally based suprapatellar synovial flap was mobilised and reflected distally to cover the trochlear groove and contain the graft material, forming a biological interposition layer (Figure 3). Patellar tracking over the interposed tissue was satisfactory without impingement or instability. The joint capsule and skin were closed in layers.

**Figure 4:**
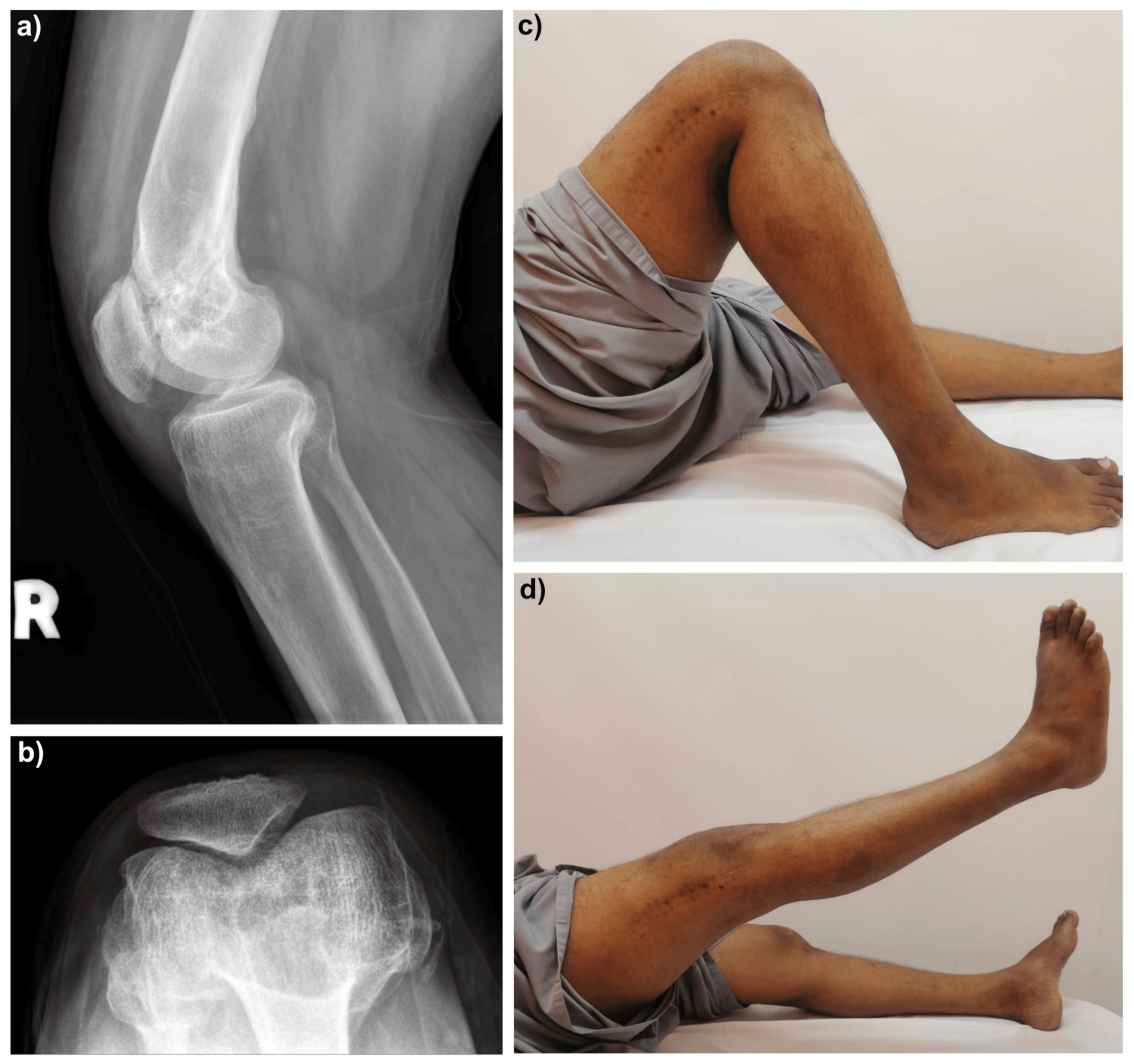
Two-year post-operative radiographic and clinical assessment of the right knee (a) Lateral radiograph showing restoration of patellofemoral joint alignment (b) Merchant view demonstrating a congruent patellofemoral articulation with maintained joint space (c) Clinical photograph illustrating active knee flexion up to 110 degrees (d) Clinical photograph demonstrating full active knee extension

Postoperatively, the patient commenced continuous passive motion and was allowed protected weight-bearing. At six weeks, he reported significant pain relief and resumed full weight-bearing ambulation. Follow-up radiographs demonstrated progressive ossification of the trochlear defect and a congruent patellofemoral articulation (Figure 4). At two-year follow-up, he remained asymptomatic and maintained functional improvement, with a ROM of 0-110 degrees, independent stair climbing and return to work without the need for analgesics. His Knee Society Score (KSS) improved significantly, with satisfaction increasing from 4 to 16 and function from 8 to 45.

## Discussion

Surgical management of isolated patellofemoral joint osteoarthritis (PFJ OA), particularly in young and active individuals, presents a significant clinical challenge due to the unique biomechanics and morphological variability of the patellofemoral articulation. High joint contact pressures during flexion and the dynamic nature of load transmission across the joint often exacerbate degenerative changes and limit the longevity of conventional reconstructive procedures [2,3].

In early-stage PFJ OA, procedures such as soft tissue realignment, chondroplasty, or focal cartilage restoration may offer symptomatic relief. However, their success is largely limited to small, contained lesions in morphologically normal joints [2]. For advanced disease with extensive cartilage loss or complex post-traumatic deformity, prosthetic options are typically considered. Total knee arthroplasty (TKA), while reliable and effective, is often regarded as excessive in younger patients due to concerns about implant longevity and the loss of native joint mechanics. Patellofemoral arthroplasty (PFA) represents a less invasive alternative that preserves tibiofemoral articulation; however, its outcomes in post-traumatic scenarios remain inconsistent. High rates of revision, mal-tracking, and progression to tibiofemoral osteoarthritis have been reported, especially when joint anatomy is distorted [4].

In the presented case, both TKA and PFA were suboptimal choices. The patient’s young age, high functional demand, and the presence of a large, irregular trochlear bone defect complicate the feasibility of implant fixation and stability. Additionally, the relatively thin patella (18 mm) raised concerns about potential periprosthetic fracture with resurfacing procedures.

Osteochondral allograft transplantation is a treatment option for large areas of cartilage loss; however, it is not readily available in our region. It is technically demanding, costly, and carries risks of immunogenicity and infection [8,9]. Bipolar defects, as in our patient, have reported failure rates of up to 50% [10]. Furthermore, the large trochlear defect would require a shell allograft, which has a survivorship of only 65.8% at five years and 37% at ten years [11]. Graft incorporation may also be compromised by mechanical instability and limited host bone contact due to the size and depth of the trochlear bone defect [12].

Interposition arthroplasty offers a compelling, biologically based alternative for joint preservation in such complex cases. By placing a soft tissue graft between articulating surfaces, the technique creates a fibrocartilaginous pseudo-joint that can reduce pain, restore function, and delay the need for prosthetic replacement. Although its use has declined with the rise of joint arthroplasty, interposition arthroplasty has demonstrated favourable outcomes in other anatomically challenging joints such as the elbow, shoulder, and thumb carpometacarpal joint [6,7].

A variety of graft materials have been employed, including autografts (e.g., fascia lata, quadriceps tendon, synovium), allografts, and synthetic substitutes [7,8,13,14]. Autologous options are generally preferred due to their biocompatibility and reduced risk of immune reaction or foreign body response. In this case, a proximally based suprapatellar synovial flap was selected as the interposition material. It offered several advantages: it was locally available, large enough to cover the trochlear defect, spared donor site morbidity, and had become thickened due to trauma, rendering it robust and suitable for interposition.

Anchoring the synovial flap to the trochlear surface achieved two critical objectives: biological resurfacing of the degenerated femoral articular surface and containment of the demineralised bone graft within the defect. This approach resembles the technique used in complex revision TKA described by Abdel et al., who used soft tissue coverage to restore the patellar defect during bone grafting [15]. To our knowledge, this represents the first reported case of using a synovial flap anchored to the trochlea for interposition arthroplasty.

While the fibrocartilage that forms over time may not replicate the biomechanical properties of hyaline cartilage, clinical studies have shown that it provides sufficient durability to reduce symptoms and improve function [8]. In our patient, the procedure allowed restoration of joint congruency, significant pain relief, and return to daily activities without further intervention, outcomes maintained over a two-year follow-up period.

Pre-operative planning for biological interposition arthroplasty of the patellofemoral joint is limited by the difficulty in accurately determining the extent of cartilage damage and identifying suitable biological flaps. Magnetic resonance imaging (MRI) remains the cornerstone for cartilage and synovial assessment, yet differentiating thin residual cartilage from fibrocartilage or hypertrophic synovium can be challenging. Imaging often underestimates the true extent of cartilage loss and subchondral bone changes, which can complicate graft sizing and fixation planning. Identifying suitable synovial flaps can be aided by contrast-enhanced MRI, which distinguishes synovium from the similar-appearing joint fluid, but at the expense of longer scan time and potential contrast-related adverse reactions [16]. Advances in high-field, high-resolution conventional MRI have improved the reliability of synovial thickness measurement without contrast administration. On multiaxial proton-density and T2-weighted images, hypertrophied synovium appears with lower signal intensity than joint fluid [17]. Nevertheless, quantitative pre-operative assessment of viable synovial tissue for flap harvesting remains limited and the specificity of non-contrast MRI for this purpose is yet to be established. Intraoperative findings may still reveal inadequate or unsuitable tissue for grafting. Therefore, surgeons must plan with flexibility, anticipating the need for alternative graft sources and intraoperative modification of the procedure.

## Conclusions

Interposition arthroplasty remains a valuable, albeit underutilised, joint-preserving option in the management of isolated patellofemoral arthritis, particularly in young, active patients with complex post-traumatic anatomy where conventional arthroplasty is either unsuitable or carries a high risk of failure. In this case, the use of a suprapatellar synovial flap provided an effective biological resurfacing material that simultaneously addressed a large trochlear bone defect and preserved native joint function. The favourable clinical and functional outcomes observed at two-year follow-up support the viability of this technique as both a definitive and temporising solution. Interposition arthroplasty, when carefully selected and appropriately executed, offers a low-cost, biologically sound alternative that maintains future surgical options while restoring function and improving quality of life.
